# Isolation and Characterization of Two Kinds of Stem Cells from the Same Human Skin Back Sample with Therapeutic Potential in Spinal Cord Injury

**DOI:** 10.1371/journal.pone.0050222

**Published:** 2012-11-30

**Authors:** Zhaowen Zong, Nan Li, Xinze Ran, Yongping Su, Yue Shen, Chun-meng Shi, Tian-min Cheng

**Affiliations:** 1 State Key Laboratory of Trauma, Burn and Combined Injury, Department of Trauma Surgery, Daping Hospital, Third Military Medical University, ChongQing, China; 2 State Key Laboratory of Trauma, Burns and Combined Injury, Institute of Combined Injury, School of Military Preventive Medicine, Third Military Medical University, ChongQing, China; Oregon Health & Science University, United States of America

## Abstract

**Backgrounds and Objective:**

Spinal cord injury remains to be a challenge to clinicians and it is attractive to employ autologous adult stem cell transplantation in its treatment, however, how to harvest cells with therapeutic potential easily and how to get enough number of cells for transplantation are challenging issues. In the present study, we aimed to isolate skin-derived precursors (SKPs) and dermal multipotent stem cells (dMSCs) simultaneously from single human skin samples from patients with paraplegia.

**Methods:**

Dissociated cells were initially generated from the dermal layer of skin samples from patients with paraplegia and cultured in SKPs proliferation medium. Four hours later, many cells adhered to the base of the flask. The suspended cells were then transferred to another flask for further culture as SKPs, while the adherent cells were cultured in dMSCs proliferation medium. Twenty-four hours later, the adherent cells were harvested and single-cell colonies were generated using serial dilution method. [^3^H]thymidine incorporation assay, microchemotaxis Transwell chambers assay, RT-PCR and fluorescent immunocytochemistry were employed to examine the characterizations of the isolated cells.

**Results:**

SKPs and dMSCs were isolated simultaneously from a single skin sample. SKPs and dMSCs differed in several respects, including in terms of intermediate protein expression, proliferation capacities, and differentiation tendencies towards mesodermal and neural progenies. However, both SKPs and dMSCs showed high rates of differentiation into neurons and Schwann cells under appropriate inducing conditions. dMSCs isolated by this method showed no overt differences from dMSCs isolated by routine methods.

**Conclusions:**

Two kinds of stem cells, namely SKPs and dMSCs, can be isolated simultaneously from individual human skin sample from paraplegia patients. Both of them show ability to differentiate into neural cells under proper inducing conditions, indicating their potential for the treatment of spinal cord injury patients by autologous cell transplantation.

## Introduction

Spinal cord injury (SCI) remains a great challenge to clinicians, and stem cell transplantation represents an attractive option for its treatment [1]. Although embryonic stem cells, fetal tissue-derived neural stem cells, inducible pluripotent stem cells and adult neural stem cells are candidate sources for transplantation, their use is associated with both ethical and clinical issues, including the need for immune suppression and their limited supplies [1], [2]. The discovery of precursor cells or stem cells with neural potential in adult tissues such as skin [3]–[5], bone marrow [6], gut [7], and pancreas [8] provides a potential adult stem cell source for neural transplantation.

The skin has many advantages as a potential stem cell source for the practical therapeutic treatment of SCI, including its abundance, easy accessibility, high self-renewal ability, and the possibility of achieving autologous transplantation [3]–[5], [9]. To date, many kinds of stem or precursor cells have been found in skin, including epidermal stem cells, dermal multipotent mesenchymal stem cells (dMSCs), skin-derived precursors (SKPs) and dermal fibroblast [3]–[5], [9]–[11]. Some of these cells possess the potential to differentiate into neurons and glial cells under proper inducing conditions, and have shown the ability to repair SCI after transplantation [5], [9].

Obtaining sufficient numbers of stem cells for transplantation remains a key issue in stem cell transplantation. Adjusting culture conditions has been a widely and successfully employed method for expanding stem cells [12], [13]. However, to obtain two or more kinds of stem cells from a single sample simultaneously might provide another promising option, especially in the case of human samples, when only limited amounts of sample can be obtained.

In the present study, we aimed to isolate SKPs and dMSCs simultaneously from single human back skin samples from patients with paraplegia. Biological properties of SKPs and dMSCs isolated in this way was assessed, with special emphasis on their differentiation potential toward neural cells to see whether they could be used as candidates for cell transplantation in SCI repair.

## Materials and Methods

The experimental protocol was reviewed and approved by the Ethical Committee of the Daping Hospital, Third Military Medical University, P. R. China.

### Isolation of SKPs and dMSCs from individual human back skin samples

Back skin samples (1 cm×5 cm) were obtained from six patients with thoracic SCIs, during decompression and internal fixation surgery. There were five males and one female, aged 27–52 years (mean 40.3 years). Incision healing was not affected by this procedure. All patients signed informed consent forms.

Skin tissues were washed with cold phosphate-buffered saline (PBS, Boster, Wu Han, China, China), cut into 4–6-mm^2^ pieces and incubated in trypsin (Invitrogen, Carlsbad, CA) overnight at 4°C. Epidermis was manually removed and the dermis was cut into 1-mm^3^ pieces and incubated in trypsin for 30 minutes at 37°C. Then 10% fetal bovine serum (FBS, Invitrogen, Carlsbad, CA) was added to inhibit the trypsin, and the dermis was dissociated by flushing with a 2-ml pipette, until the tissue could be broken down no further. The cell suspension was centrifuged at 1,000 rpm for 5 minutes. The cell pellet was collected and re-suspended in DMEM/F12 (3∶1) (Invitrogen) and passed through a nylon mesh to remove cellular debris. The strained cell suspension was centrifuged and the cell pellet was used in the following procedures.

### Procedure 1: Simultaneous isolation of SKPs and dMSCs from the same sample

The cell pellet was re-suspended in SKPs proliferation medium consisting of DMEM/F12 (3∶1, Invitrogen) containing 40 ng/ml fibroblast growth factor-2 (FGF2, BD Biosciences, San Diego) and 20 ng/ml epidermal growth factor (EGF, BD Biosciences), similar to the procedure reported by Toma et al [3]. Cells were then transferred to a 25-cm^2^ tissue culture flask. Many cells had adhered to the bottom of the flask after 4 hours, and the suspended cells were then removed and transferred to another flask, and further cultured in SKPs proliferation medium. The remaining adherent cells were cultured in dMSCs proliferation medium, consisting of Iscove's modified Dulbecco's medium (IMDM) (Invitrogen) containing 10% FBS. Twenty-four hours later, the adherent cells were harvested and single-cell colonies were generated by serial dilution, as previously reported [4]. Briefly, the cells were serially diluted into IMDM containing 10% FBS and seeded in 96-well plastic culture plates. Each well contained 200 µl IMDM plus 10% FBS and was assessed microscopically. Wells containing more than one cell were discarded, and only wells containing a single cell were used. A cell population that produced cell clones of more than 20 cells was considered to be a colony and was passaged and expanded as usual. dMSCs isolated in this way were named “new dMSCs” (N-dMSCs).

### Procedure 2: Isolation of dMSCs by routine method

Following previously reported methods [4], [14], the cell pellet was suspended in 10 ml IMDM containing 10% FBS and transferred to a flask. Four hours later, many cells had adhered to the bottom of the flask and the medium containing the suspended cells was discarded. The adherent cells were further cultured in IMDM/10% FBS for 12 hours, and dMSCs were then isolated using the serial dilution method, as described in procedure 1. dMSCs isolated in this way were named “routine dMSCs” (R-dMSCs).

### Cell proliferation assay

The cell proliferation rate was measured by [^3^H] thymidine incorporation assay [15]. Briefly, about 5×10^4^ cells per well were seeded in 24-well plates. [^3^H]thymidine (Chinese Academy of Sciences Institute of Nuclear Research, Shanghai, China) at a final concentration of 2 µCi/ml was added to the cell cultures and the cells were cultured for 3 days. The medium was then aspirated, the cells were washed three times with ice-cold PBS, precipitated with ice-cold 15% trichloroacetic acid (Chuan Dong Chemical group, Chongqing, China) overnight, lysed with 1 N NaOH (Chuan Dong Chemical group), and incubated at room temperature for 2 hours. Afterwards, 50 µl of the lysate was transferred to 96-well plates in duplicate, and 200 µl of scintillation solution was added. The plates were put on a shaker for 4 hours and the amount of radioactivity was counted using an LS6500 Multi-purpose Scintillation Counter (Beckman Coulter, Inc., Brea, CA).

### Migration ability assay

The migration abilities of dMSCs and SKPs were evaluated using 12-well microchemotaxis Transwell chambers (Corning Costar, Cambridge, MA), according to the manufacturer's instructions [14]. The filters were placed in Boyden chambers. The upper compartment contained 1×10^5^ cells suspended in appropriate medium, and the lower compartment contained 25 µg/ml recombinant human stromal-derived factor (rhSDF-1, Peprotech, Rocky Hill, NJ) diluted with serum-free DMEM/F-12 as a chemoattractant. After 48 hours of incubation at 37°C, cells on the top side of the filter were removed, and cells that had migrated through the filter and attached to the bottom of the membrane were fixed and stained using hematoxylin staining. Cells that had migrated though the pores and adhered to the lower surface of the membrane were analyzed under high-power (×400) light microscopy and counted in five random high-power fields (HPF). Experiments were performed in triplicate, and data were expressed as means of the numbers of cells per HPF (cells/HPF) ± standard error (SE).

### In vitro differentiation assays

Cell suspensions of dMSCs and SKPs were plated on eight-well chamber slides at a density of 3×10^4^ cells/cm^2^. dMSCs and SKPs were cultured for 1 day in IMDM supplemented with 10% FBS and DMEM/F12 (3∶1) supplemented with 5% FBS, respectively, and then transferred to differentiation medium [3], [4], [13], [16]. Adipogenic medium consisted of basal medium supplemented with 0.5 mM isobutyl-methylxanthine, 1 µM dexamethasone, 10 µM insulin, and 200 µM indomethacin (all from Sigma-Aldrich, St. Louis, MO). Myogenic medium consisted of basal medium supplemented with 10 ng/ml transforming growth factor-beta-1 (Peprotech). Neuronal differentiation medium consisted of basal medium supplemented with 10 ng/ml of brain-derived neurotrophic factor and neurotrophin-3 (both from BD Bioscience) and 6 ng/ml retinoic acid (Sigma-Aldrich). Schwann cell differentiation medium consisted of basal medium supplemented with 10 ng/ml heregulin β (R&D Systems Inc., Minneapolis, MN).

Medium was changed every 3 days in all differentiation assays, and cells were fixed for histochemical staining after 14 days of differentiation. For adipogenic differentiation, cells were stained with Oil Red O reagent (Chuan Dong Chemical group) to examine oil droplet generation in the cytoplasm. Briefly, the cells were incubated in 2% (wt/vol) Oil Red O reagent for 5 minutes at room temperature. Excess stain was removed by 70% ethanol, followed by several washes in distilled water. The cells were counterstained for 2 minutes with hematoxylin (Chuan Dong Chemical group). Myogenic and neurogenic differentiation were examined by fluorescence immunocytochemistry and reverse transcription-polymerase chain reaction (RT-PCR), as described below.

### RT-PCR

Total RNA was extracted from N-dMSCs, R-dMSCs and SKPs 14 days after induction using TripureTM (Promega Corp., Madison, WI), according to the manufacturer's instructions, and was quantified by spectrophotometry. cDNA was prepared from 1.0 µg RNA. Primers to amplify human peroxisome proliferator-activated receptor γ 2 (pparγ2) (forward primer: 5′-TGAACGACCAAGTAACTCTCC-3′; reverse primer: 5′-CTCATGTCTGTCTCCGTCTTC-3′; product: 460 bp) and α-smooth muscle actin (α-SMA) (forward primer: 5′-GGTGATGGTGGGAATGGG-3′; reverse primer: 5′-GCAGGGTGGGATGCTCTT-5′; product: 188 bp) were used to detect pparγ2 and α-SMA. Primers (forward primer: 5′-TCA TCAGCGAAAGTGGAAA-3′; reverse primer: 5′-TGTCTGTCTCACAAG GGAAGT-3′) to detect hypoxanthine guanine phosphoribosyl transferase (HGPRT) were added to each reaction tube as an internal control. pparγ2 and α-SMA bands were quantified by densitometry and expressed as a proportion relative to the average value of HPGRT. All the primers used were sythesized by Sangon Biotech (Shang Hai, China)

### Fluorescent immunocytochemistry and quantification

Fluorescent immunocytochemical analysis of SKPs, dMSCs and differentiating cells was performed. In the case of SKPs, 100 µl of medium containing suspended spheres was removed from a flask, spun down onto coated slides and air-dried for 5 minutes for further analysis. In the case of dMSCs, cells were plated on polylysine-coated coverslips. When cells reached 80% confluence, dMSCs were fixed with 95% ethanol (Chuan Dong Chemical group) in PBS for 20 minutes. The differentiated cells were fixed using the same method as for dMSCs.

The above cells were then washed three times with PBS and blocked for 1 hour at room temperature with PBS containing 10% BSA (Boster). Primary antibodies were added and incubated overnight at 4°C. Primary antibody was removed, cells were washed three times with PBS, and the appropriate secondary antibody conjugated to PE or FITC was added in PBS for 1 hour at room temperature. Cells were washed three times with PBS, stained with Hoechst 33258 (Boster), and visualized under a fluorescence reverse microscope. Primary antibodies used were βIII-tubulin monoclonal (1∶100), cytokeratin polyclonal, nestin polyclonal, fibronectin polyclonal, 2′,3′-cyclic nucleotide 3′-phosphodiesterase (CNPase) polyclonal, glial fibrillary acidic protein (GFAP) monoclonal, and vimentin polyclonal (1∶200). All primary and secondary antibodies were from Santa Cruz Biotechnology.

To obtain an estimate of the percentage of cells expressing a given marker protein, at least five fields were photographed for any given experiment, and the number of positive cells was determined relative to the total number of Hoechst-labeled nuclei. The mean and standard deviation were determined based on the results of three different tests.

### Statistical analysis

All data were expressed as means ± SE. Statistical significance was evaluated using unpaired Student's *t*-tests for comparisons between two groups, or by analysis of variance for multiple comparisons. A value of *P*<0.05 was considered significant.

## Results

### dMSCs and SKPs could be isolated simultaneously from one skin sample and showed no overt differences with dMSCs and SKPs isolated in routine way

**Figure 1:**
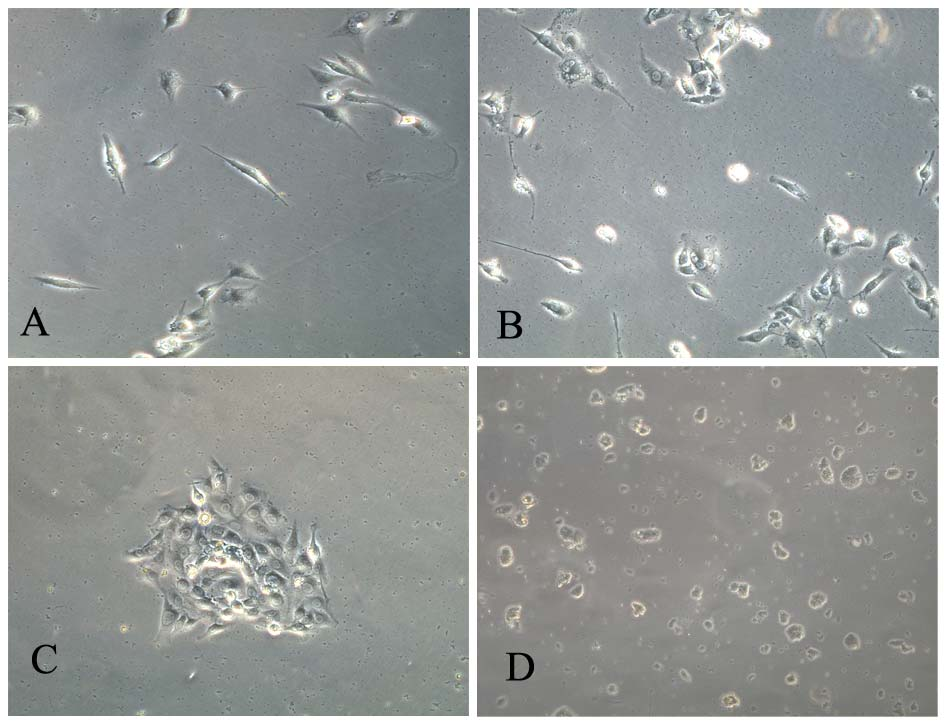
Isolation of N-dMSCs, R-dMSCs and SKPs. (A): Many cells adhered to the bottom of flask 4 hours after dissociated dermal cells were cultured in SKPs proliferation medium (×200). (B): Colony formed by serial dilution method in the process isolating N-dMSCs (×200). (C): More cells adhered to the bottom of flask 4 hours after dissociated dermal cells were culture in IMDM/10%FBS when compared with those cultured in SKPs proliferation medium (×200). (D) About 7 days after dissociated dermal cells were cultured in SKPs proliferation medium, many floating spheres formed (×100).

Dermal cells were firstly cultured in SKP proliferation medium , and 4 hours later cells remaining in suspension were transferred to another flask and further cultured in SKPs proliferation medium to isolate SKPs. The adherent cells were cultured in dMSCs proliferation medium to isolate dMSCs. The adherent cells demonstrated a flattened and fibroblast-like morphology after 24 hours (Fig. 1A). The cells were then collected and dMSCs were isolated using a single-cell clongenic culture method. A total of 384 single-cell cultures were observed for each sample and a total of 10 colonies formed, with an average of 1.66 colonies per sample (Fig. 1B). The colonies were expanded for subsequent analysis. More cells adhered to the bottom of the flask after 4 hours in primary culture using the routine dMSCs isolation method (Fig. 1C); however, the colony-forming rates were similar (1.83 colonies per sample).

**Figure 2:**
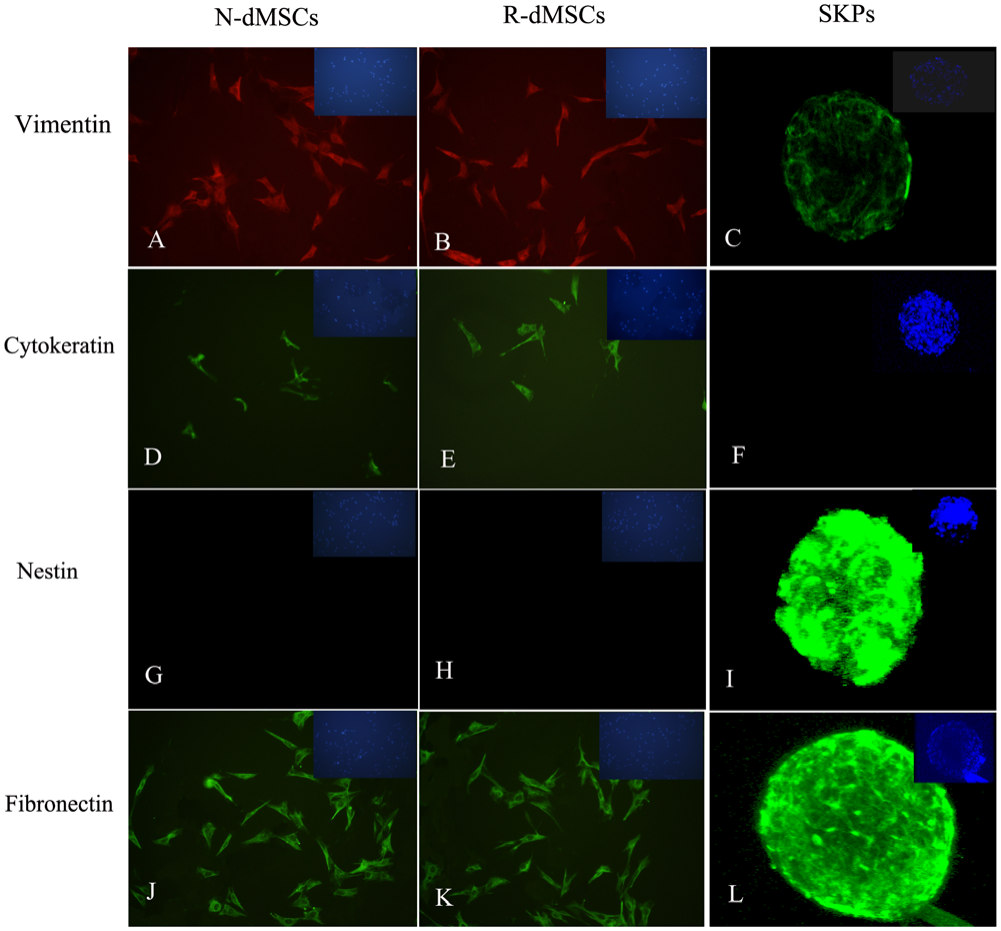
Characterization of N-dMSCs, R-dMSCs and SKPs by fluorescent immunocytochemistry. Top right panel in each picture showed the nuclei counterstained by Hoechst 33258 (×200).

**Figure 3:**
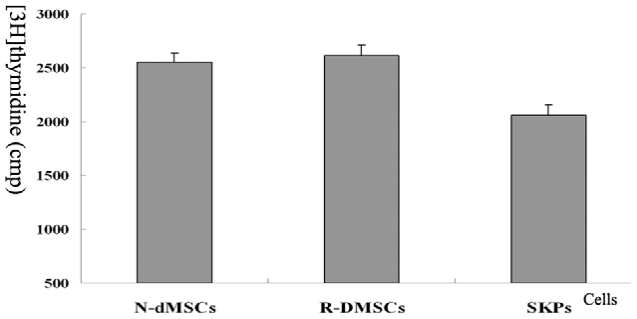
Proliferation characterization of N-dMSCs, R-dMSCs and SKPs as determined by [^3^H] thymidine incorporation assay. *: *P*<0.05, compared with the value of N-dMSCs and R-dMSCs.

Fluorescence immunocytochemistry examination showed that both R-dMSCs and N-dMSCs were positive for vimentin and fibronectin, weakly positive for cytokeratin, and negative for nestin (Fig. 2). There was no significant difference in proliferation capacity between N-dMSCs and R-dMSCs, as determined by [^3^H]thymidine incorporation assay (*P*>0.05) (Fig. 3).

**Figure 4:**
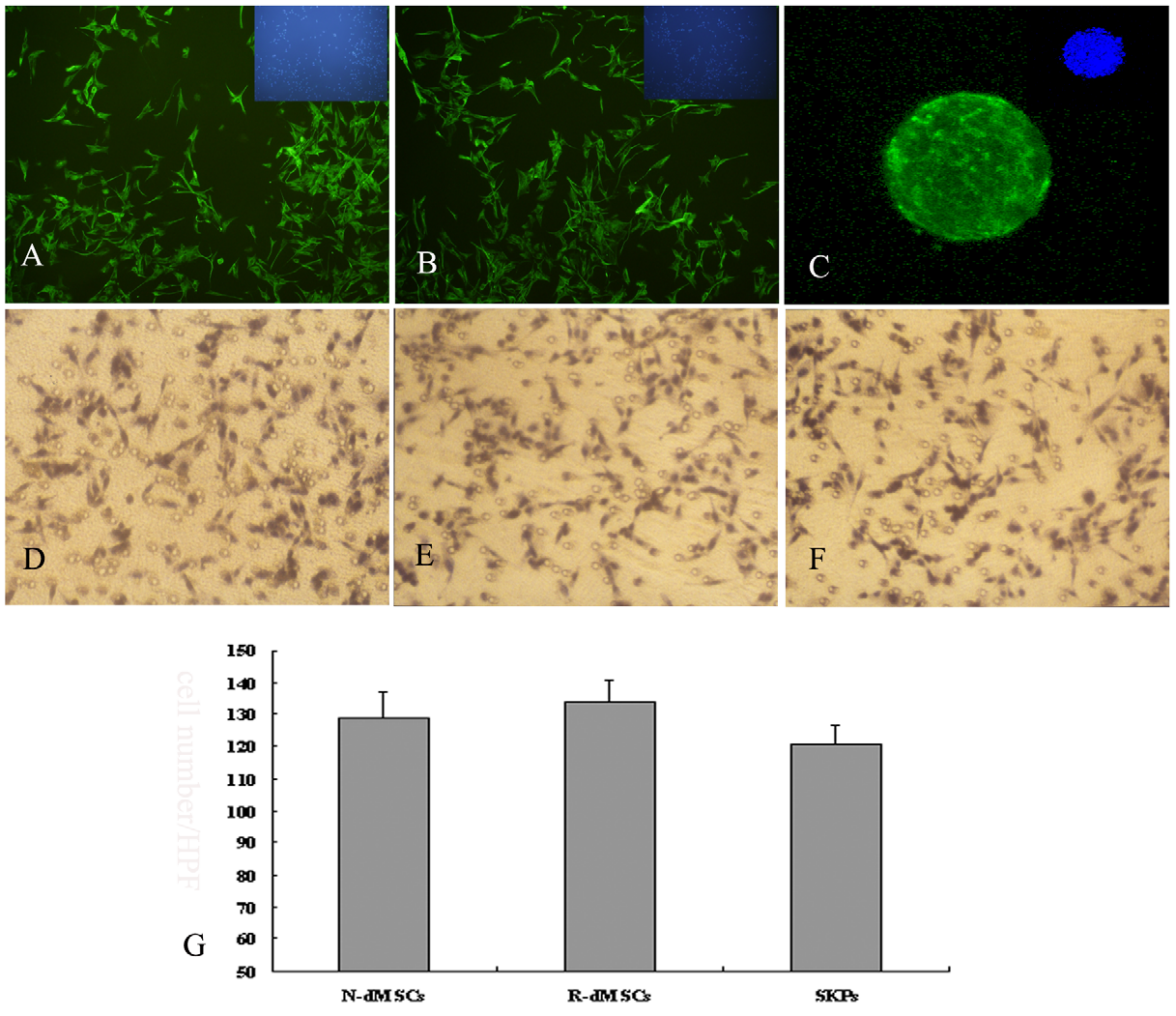
Migration characterization assay. (A–C): Fluorescent immunocytochemistry examination showed that almost all the cells of N-dMSCs, R-dMSCs and SKPs were positive to CXCR4 (×200). (D–E): N-dMSCs, R-dMSCs and SKPs that migrated to the lower chamber under the chemotactic effect of rhSDF-1, respectively (×200). F: The number of cells that migrated to the lower chamber under the chemotactic effect of rhSDF-1.

We tested the migratory ability of dMSCs and SKPs by observing the reaction of cells to rhSDF-1 since SDF-1 is a powerful chemoattractant for many kinds of stem cells [17]–[19]. As shown in Fig. 4, both N-dMSCs and R-dMSCs expressed CXCR4, and there was no significant difference in the number of cells migrating to the lower chamber between N-dMSCs and R-dMSCs, indicating no obvious differences in migration ability towards rhSDF-1 (Fig. 4).

**Figure 5:**
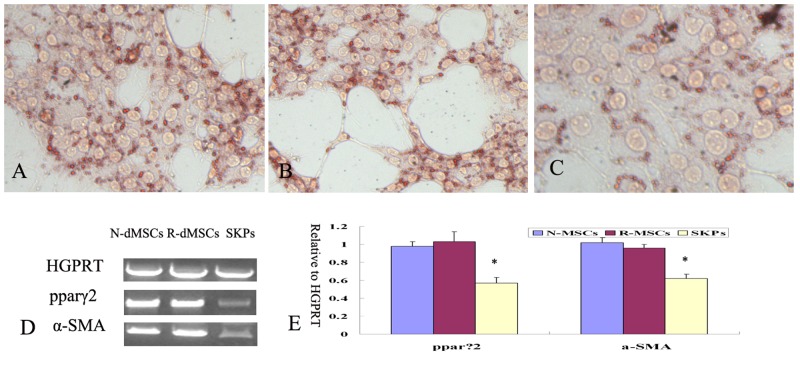
Differentiation of N-dMSCs, R-dMSCs and SKPs toward mesodermal progeny. (A–C): Oil droplet (arrows) formed in N-dMSCs, R-dMSCs and SKPs respectively as stained by Oil Red O (×200). (D): RT-PCR examination showed that the expression level of pparγ2 and α-SMA were stronger in induced N-dMSCs and R-dMSCs than that those in induced SKPs. (E): The relative value of expression level of pparγ2 and α-SMA in cells. *: *P*<0.05, compared with the value of N-dMSCs and R-dMSCs.

**Figure 6:**
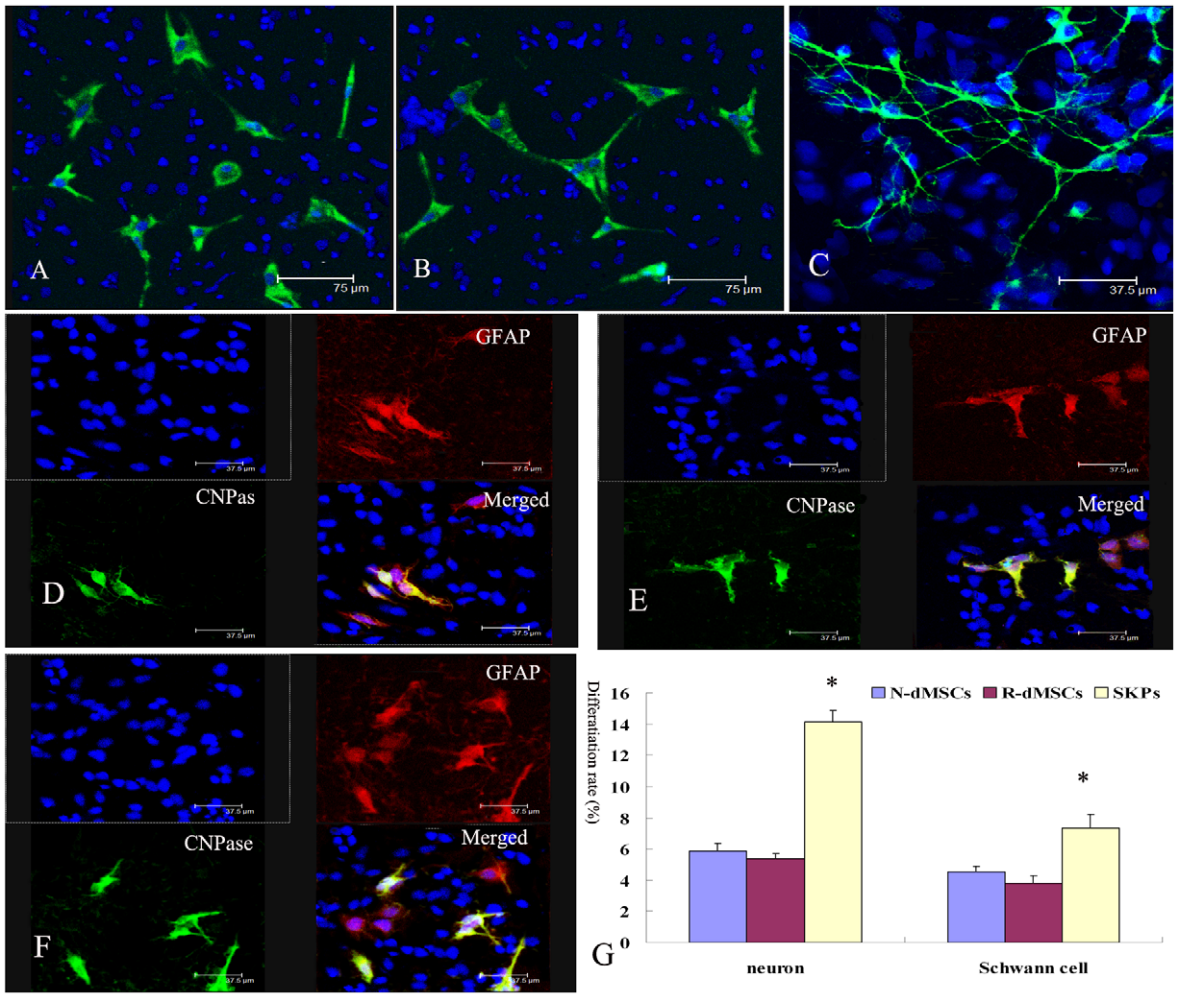
Differentiation of N-dMSCs, R-dMSCs and SKPs toward neural progeny. (A–C): Induction of N-dMSCs, R-dMSCs and SKPs toward βIII-tubulin positive cells. Arrow showed the complex morphology of dentrite in SKPs-induced neurons. (D–F): Induction of N-dMSCs, R-dMSCs and SKPs toward glia cells. Arrowheads indicate cells that only produce GFAP, and arrows indicate cells producing both GFAP and CNPase. (G) Differentiation rates of N-dMSCs, R-dMSCs and SKPs to potential neuron and Schwann cells. *: *P*<0.05, compared with the value of N-dMSCs and R-dMSCs.

Small oil droplets appeared gradually in the cytoplasm of N-dMSCs and R-dMSCs about 7–10 days after induction. Both N-dMSCs and R-dMSCs stained positively stained with Oil Red O about 14 days after induction. The extent and volume of the oil droplets were similar between N-dMSCs and R-dMSCs (Fig. 5A,B). Under appropriate induction conditions, both N-dMSCs and R-dMSCs were able to differentiate into potential neurons (βIII-tubulin-positive cells) and Schwann cells (cells positive for both CNPase and GFAP) (Fig. 6). There were no significant differences between the rates of differentiation of N-dMSCs and R-dMSCs into potential neurons and Schwann cells (the differentiation rate into potential neurons was 5.85%±0.52% in N-dMSCs and 5.37%±0.34% in R-dMSCs respectively; the differentiation rate into potential Schwann cells was 4.55%±0.32% in N-dMSCs and 3.78%±0.51% in R-dMSCs respectively, *P*>0.05, Fig. 6G).

Cells remaining in suspension after 4 hours of initial culture were transferred to another flask and further cultured to isolate SKPs. More cells adhered to the bottom of the flask, but after about 7 days, some floating spheres were found (Fig. 1). The cells were then further cultured and passaged as usual.

Fluorescence immunocytochemistry examination revealed that the intermediate protein expression patterns of SKPs isolated this way were the same as reported by Toma, et al [3], [20], [21], namely positive for vimentin, nestin and fibronectin, and negative for cytokeratin (Fig. 2). [^3^H]thymidine incorporation and chemotactic assay showed that SKPs could proliferate and migrate efficiently (Fig. 3 and Fig. 4). In vitro differentiation assays demonstrated that SKPs could differentiate into neurons, Schwann cells and adipocytes under appropriate inducing conditions (Fig. 5 and Fig. 6). Taken together, these data indicate that SKPs could be isolated simultaneously along with dMSCs, and their properties were not influenced.

### Differences between dMSCs and SKPs

Because dMSCs and SKPs were isolated from the same skin sample, it was necessary to determine if they were the same type of cells isolated under different culture conditions.

Firstly fluorescence immunocytochemistry revealed that dMSCs and SKPs have different kinds of intermediate protein expression. As shown in Fig. 2, dMSCs were positive for vimentin, and negative for nestin, indicating that their mesenchymal origin. SKPs, however, were negative for cytokeratin, but positive for nestin and fibronectin, which are typical antigen markers of neural crest-derived cells in skin.

dMSCs showed higher proliferative activity than SKPs (*P*<0.05) (Fig. 3), but similar migratory abilities in response to the chemotactic influence of SDF-1 (Fig. 4).

Under appropriate inducing conditions, both dMSCs and SKPs were able to differentiate into mesodermal progeny and neural cells, however, the extents and tendencies differed. Fourteen days after induction, oil droplets were seen in SKPs, but the number and volume of the droplets were smaller than in N-dMSCs and R-dMSCs (Fig. 5). pparγ2 is a transcription factor that regulates adipogenesis and has therefore been used to reflect the level of adipogenic differentiation. The results of RT-PCR showed that the level of pparγ2 mRNA expression in SKPs was weaker than that in N-dMSCs and R-dMSCs (Fig. 5). Similar results were obtained regarding differentiation into smooth muscle cells (Fig. 5).

Fourteen days after induction, N-dMSCs and R-dMSCs could differentiate into potential neurons and Schwann cells, but the differentiation rates were lower than in SKPs (the differentiation rate into potential neurons was 5.85%±0.52% in N-dMSCs, 5.37%±0 .34% in R-dMSCs , and 14.12%±0.74% in SKPs respectively; while the differentiation rate into potential Schwann cells was 4.55%±0.32% in N-dMSCs, 3.78%±0.51% in R-dMSCs , and 7.35%±0.85% in SKPs respectively, *P*<0.05, Fig. 6G). In addition, the neurons generated from SKPs were morphologically more complex than those generated from N-dMSCs and R-dMSCs (Fig. 6A–C). Thus SKPs and dMSCs showed different tendencies to differentiate into mesodermal and neural progeny.

## Discussion

Skin represents a promising candidate for stem cell transplantation, in light of its easy accessibility, high self-renewal ability, and the possibility to achieve autologous transplantation. To date, many kinds of stem or precursor cells have been isolated from skin, some of which show potent capacities to differentiate into neural or glial cells under appropriate conditions, thus making them promising candidates for cell transplantation for the repair of SCI. SKPs isolated from the dermal layer of skin generated Schwann cells under the induction of neural crest cues such as neuregulins, and could myelinate brain axons when transplanted into the demyelinated brain of neonatal shiverer mice [20]. SKPs also promoted remyelination and functional recovery after contusion spinal cord injury in rats [21]. dMSCs represent another type of stem cells found in the dermis, and are also able to differentiate into neurons and glial cells, and show an ability to repair SCI after transplantation [5]. Thus both SKPs and dMSCs represent promising treatments for SCI.

In the present study, dMSCs and SKPs were isolated simultaneously from the same back skin sample from patients with paraplegia when SKP proliferation medium (DMEM/F12 containing 40 ng/ml FGF2 and 20 ng/ml EGF) was used during the initial stage. The isolated SKPs showed similar intermediate protein expression pattern and differentiation potential as reported in previous studies [3], [20], [21]. Cells that adhered to the bottom of the flask during the initial culture were used to isolate N-dMSCs. N-dMSCs and R-dMSCs showed no overt differences in intermediate protein expression, growth, migration or differentiation properties. The only difference between the isolation procedures for N-dMSCs and R-dMSCs was that N-dMSCs were cultured for the initial 4 hours in SKP proliferation medium, while R-dMSCs were cultured in dMSC proliferation medium during this period. The results showed that culture in SKP proliferation medium for the first 4 hours had no effect on the properties of dMSCs. One thing should be mentioned is that dMSCs and SKPs could only be isolated simultaneously from the same skin sample when SKP proliferation medium was used during the initial stage but not dMSCs proliferation medium (IMDM/10%FBS). When dMSCs proliferation medium was initially used, more cells adhered to the bottom of flask and less cells kept afloat when compared with that when SKPs proliferation medium was used during the initial stage. Four hours later, the suspended cells were collected and transferred to another flask and cultured in SKPs proliferation medium. However, no obvious floating sphere formed even 10 days later (data not shown). Until now, the definite reasons for this difference are unknown to us. Overall, these results indicate that SKPs and dMSCs can be simultaneously obtained from the same back skin samples from patients with paraplegia, and both were able to efficiently differentiate into neurons and Schwann cells.

In contrast, N-dMSCs and SKPs differed from each other in many ways, such as the conditions required to generate them, their morphologies (N-dMSCs were adherent, fibroblast-like cells, while SKPs grew as spheres in suspension), their intermediate proteins expression patterns, their migratory capacities, and their potentials to differentiate towards mesodermal and neural progenies. However, these observations do not clarify the origin of or the relationship between these two types of cells. One possibility is that SKPs and dMSCs are derived from the same cell type, and that the different culture conditions result in the generation of two types of stem cells with distinct biological characteristics. The properties of stem cells have been shown to be altered by culture conditions [16], [22]. Shih [16] used DMEM culture medium containing 10% FBS, 20 ng/ml EGF and 20 ng/ml FGF2, which differed from both that of Toma et al [3] and that used in the present study, and obtained adherent, mesenchymal stem cell-like cells from human scalp tissue. The cells were adherent, like the dMSCs reported in the present study, but their tendency to differentiate towards the neural lineage was stronger than that of either SKPs or dMSCs. However, the origin of these cells has not been clarified. The second possibility is that SKPs and N-dMSCs are independently generated within the dermis during development and persist into adulthood [23]. Because the present study was based on human samples, we were unable to investigate this possibility. Data from rodents has shown that SKPs represent endogenous neural crest-related precursor cells that arise in the skin during late embryogenesis, likely via migration, and that persist in lower numbers in adult skin [24]–[26]. The origin of mesenchymal stem cells (MSCs) has not been confirmed [4], [27]–[28]. Cranial MSCs are most likely embryonically derived from the neural crest, while sub-cranial MSCs are most likely embryonically derived from the mesoderm [22].

In conclusion, although the definite origins of SKPs and N-dMSCs are not clear, they can be simultaneously isolated from the same back skin sample from patients with paraplegia. They can differentiate efficiently into potential neurons and Schwann cells under appropriate induction conditions, indicating their promising potential for the treatment of SCI patients. However, rigorous tests demonstrating the beneficial effect of such cells in SCI patients should be done before they can used t in SCI patients. Another issue is how to use these two kinds of cells more efficently: Whether to mix two types of cells before grafting or graft them separately? Whether to use undifferentiated cells, or differentiated cells? We are working on these issues now and our preliminary study showed that differentiated cells transplantation were more efficient in improving the function of contused spinal cord injury in rats (data not shown). However, more work still needed to be done before more convincing data could being obtained.
